# Association between different insulin resistance surrogates and infertility in reproductive-aged females

**DOI:** 10.1186/s12889-023-16813-2

**Published:** 2023-10-12

**Authors:** Weiting Xia, Yaoyao Cai, Sisi Zhang, Shenghao Wu

**Affiliations:** 1 Department of Gynecology, The First Affiliated Hospital of Wenzhou Medical University, Wenzhou, Zhejiang China; 2 Reproductive Medicine Center, Department of Obstetrics and Gynecology, The Second Affiliated Hospital, Yuying Children’s Hospital of Wenzhou Medical University, Wenzhou, Zhejiang 325000 China; 3 Department of Obstetrics, The First Affiliated Hospital of Wenzhou Medical University, Wenzhou, Zhejiang China; 4 Reproductive Medicine Center, Department of Obstetrics and Gynecology, The First Affiliated Hospital of Wenzhou Medical University, Wenzhou, Zhejiang China

**Keywords:** Female infertility, Insulin resistance, Insulin resistance surrogates, Triglyceride glucose-body mass index, NHANES

## Abstract

**Background:**

Obesity and metabolic syndrome are observed more frequently in infertile women, and insulin resistance (IR) is closely related to them. However, there are no studies that have examined the association between different IR surrogates and female infertility, hence we investigated the potential association between them in the general population.

**Methods:**

This was a cross-sectional study using data from the National Health and Nutrition Examination Survey (NHANES, 2013–2018). The association of different IR surrogates (HOMA-IR index, TyG index and TyG-BMI index) with female infertility was estimated by multivariable regression analysis.

**Results:**

After adjusting for confounders, the HOMA-IR index and TyG index did not show an association with female infertility, while the TyG-BMI index was found to have a positive association with female infertility (OR = 1.01, 95% CI: 1.00, 1.01; P < 0.0001), and the OR of the TyG-BMI group T3 (≥ 255.55) was significantly different compared to the group T1 (< 185.31) (OR = 3.02, 95% CI: 1.62, 5.60). Similar results were seen in most of the subgroup participants by stratified analysis (P-interaction > 0.05). However, different IR surrogates did not show variability in their ability to predict infertility [TyG-BMI: 0.68 (95% CI: 0.62, 0.74) vs. TyG: 0.62 (95% CI: 0.57, 0.68) vs. HOMA-IR: 0.65 (95% CI: 0.60, 0.71)].

**Conclusions:**

Our result suggests that high levels of TyG-BMI index were positively associated with female infertility in US reproductive-aged females.

**Supplementary Information:**

The online version contains supplementary material available at 10.1186/s12889-023-16813-2.

## Background

Infertility, defined as the inability to conceive after 12 months of unprotected sexual intercourse, is a reproductive disorder that affects both men and women [1]. It is estimated that approximately 186 million people worldwide suffer from infertility, with approximately one in seven couples in developed countries and one in four couples in developing countries [2, 3]. Among these, female infertility accounts for approximately 40% of all cases [4, 5]. Infertility has become a serious problem affecting human development, and therefore the US Centers for Disease Control and Prevention (CDC) recommends priority diagnosis and treatment of infertility [6].

Current studies suggest that the increasing age at the time of pregnancy is the most significant negative factor affecting female fertility [7], but other factors including lifestyle (nutrition, exercise, psychological stress, smoking or drinking) and environmental factors (radiation, chemicals) are considered to play increasing roles [8–10]. In addition, metabolic disorders, such as obesity and metabolic syndrome, are also common in infertile females [11, 12]. Insulin resistance (IR) has also been observed to be significantly associated with polycystic ovary syndrome (PCOS), leading to female infertility [13]. Insulin resistance is defined clinically as the inability of a known quantity of exogenous or endogenous insulin to increase glucose uptake and utilization in an individual as much as it does in a normal population. The traditional methods used to assess IR are the hyperinsulinemic-euglycemic clamp (HIEC) and homeostatic model assessment (HOMA-IR), but these methods are complex and time-consuming. In recent years, the triglyceride-glucose index (TyG index) and triglyceride-glucose-body mass index (TyG-BMI index) have been newly proposed as excellent surrogates of insulin resistance [14–17]. However, to our knowledge, there are no studies that have examined the association between different insulin resistance surrogates and female infertility.

Therefore, the objective of this study was to use a nationally representative sample of US adult females from the National Health and Nutrition Examination Survey (NHANES) to explore the potential association between different IR surrogates and female infertility, which could provide new insights into the management of female reproductive health.

## Methods

### Data sources

To provide detailed data and address critical public health issues that affecting the U.S. citizen population, the National Center for Health Statistics (NCHS) developed and conducted the National Health and Nutrition Examination Survey (NHANES). This was a large, nationally representative, cross-sectional survey conducted every two years by questionnaire and physical examination [18]. We extracted data of 14,948 female participants from the NHANES 2013–2018 database. Participants in each NHANES cycle were identified by stratified, multistage probability sampling. The Research Ethics Review Board of the National Center for Health Statistics (NCHS) approved the study of NHANES, and all study participants provided informed written consent.

### Independent and dependent variables

Blood collection was performed in the morning after fasting to collect total cholesterol, high-density lipoprotein (HDL), low-density lipoprotein (LDL), triglycerides, fasting glucose, and insulin data. The TyG index was calculated as follows: TyG = Ln[fasting triglycerides (mg/dL) × fasting glucose (mg/dL)/2] [16]. The TyG-BMI index was calculated as follows: TyG-BMI = TyG index × BMI (kg/m2) [17]. The HOMA-IR index was calculated as follows: HOMA-IR = fasting glucose (mmol/L) × fasting insulin (µU/mL)/22.5 [19]. IR was defined according to the Homeostatic Model Assessment, defined as HOMA-IR ≥ 2.2 [20].

The dependent variable of infertility derived from each woman’s self-report from the Reproductive Health Questionnaire. In this questionnaire, the investigators asked the question “Tried for a year to become pregnant?“. An answer of “yes” indicates an “infertile” case, a negative answer indicates a “fertile” case.

### Other variables

Other variables in this study were collected by standard questionnaire and physical examination, including age, BMI, race, marital status, education level, household income, smoking status, and drinking status. We divided BMI into three groups: “Normal or low weight” (< 25 kg/m^2^), “Overweight” (25-29.9 kg/m^2^), and “Obesity " (≥ 30 kg/m^2^). The poverty-to-income ratio (PIR) was used as a surrogate of household income and was divided into three groups: “0-1.3 RIP,“ “1.3–3.5 RIP,“ and “> 3.5 RIP”. Drinking status was divided into four groups based on daily alcohol consumption: “None or light drinker” (≤ 1 drinks per week), “Moderate drinker” (2–8 drinks per week), “heavy drinker” (> 8 drinks per week), and “Missing data”. Personal medical history (hypertension and diabetes) was obtained from the self-report of each participant’s health questionnaire.

### Statistical analysis

In our study, continuous variables were presented as means and standard deviations, and categorical variables were presented as numbers (n) and percentages (%). The participants were divided into “Non-IR group” and “IR group” according to the HOMA-IR index, and the difference between the two groups was examined by the Chi-square test or Kruskal-Wallis H test. We used logistic regression model to assess the correlation between different IR surrogates and infertility, expressing the relationship with OR values and 95% confidence intervals (95% CI). In the analysis we developed three models, Model I without any adjustment, Model II adjusted age and Model III adjusted age, marital status, education level, LDL, hypertension and diabetes. Based on the results of these analyses, we further assessed the differences in the risk of infertility between the different TyG-BMI tertile groups (the T1 group as reference). T1 group was patients with a TyG-BMI index < 185.31, T2 group was patients with a TyG-BMI index 185.31≤ ~ <255.55 and T3 group was patients with a TyG-BMI index ≥ 255.55. In addition, we used restricted cubic spline (RCS) curves based on Model III to explore any non-linear relationship between TyG-BMI index and infertility.

Further, we performed interaction and stratified analysis according to age, race, household income, marital status, education level, BMI status, smoking status, drinking status, and histories of chronic diseases.

At last, we compared the predictability of different IR surrogates on insulin resistance and infertility by receiver operating characteristic (ROC) curves and their respective areas under the curve (AUC). Differences between AUCs were compared by the Z test.

The statistical software used to analyze all data were IBM SPSS Statistics 21 (IBM SPSS, Turkey) program and EmpowerStats software (www.empowerstats.com; X&Y solutions, Inc., Boston MA). p-value < 0.05 was considered to be statistically significant.

## Results

**Fig. 1:**
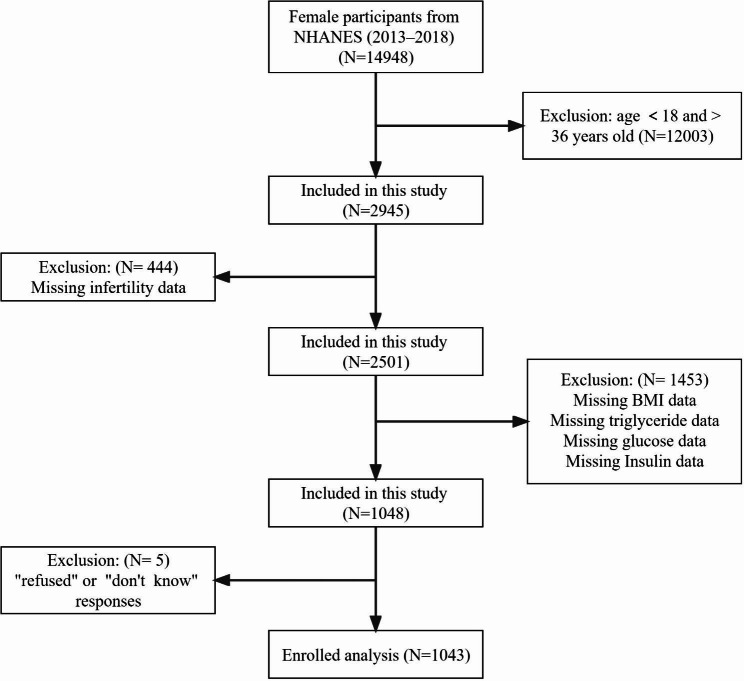
Flow chart of eligible participants’ selection

Among the 14,948 participants, we excluded 12,003 participants younger than 18 years or older than 36 years, 444 participants with missing infertility data, 1453 participants with missing BMI, triglyceride, serum insulin and fasting glucose data, and 5 participant who responded with “refused” or “unclear”, finally we included 1043 participants for the final analysis (Fig. 1).

### Baseline characteristics of study participants

Participants ultimately included in the study were divided into two groups “Non-IR group (HOMA-IR < 2.2)” and “IR group (HOMA-IR ≥ 2.2)”. Table 1 presents the baseline characteristics of the two groups. We found that participants in the IR group were more likely to be obese and to have higher LDL, triglycerides, fasting glucose, insulin, TyG index and TyG-BMI index, as well as higher risk of hypertension, diabetes and female infertility compared to the other group (P < 0.05).

**Table 1:** Demographic and clinical characteristics according to different insulin resistance groups based on HOMA-IR index

IR Groups	Non-IR	IR	P-value
**Number**	541	502	
**Age (years)**	26.90 ± 5.68	26.63 ± 5.80	0.464
**Race**			0.027
Non-Hispanic White	200 (36.97%)	153 (30.48%)	
Other Race	341 (63.03%)	349 (69.52%)	
**Marital status**			0.739
Married	169 (31.24%)	163 (32.47%)	
Other	295 (54.53%)	262 (52.19%)	
Missing data	77 (14.23%)	77 (15.34%)	
**Education level**			0.051
Less than high school	50 (9.24%)	69 (13.75%)	
High school or above	414 (76.52%)	356 (70.92%)	
Missing data	77 (14.23%)	77 (15.34%)	
**Household income**			< 0.001
0–1.3RIP	197 (36.41%)	182 (36.25%)	
> 1.3–3.5 RIP	181 (33.46%)	195 (38.84%)	
> 3.5 RIP	133 (24.58%)	79 (15.74%)	
Missing data	30 (5.55%)	46 (9.16%)	
**BMI status**			< 0.001
Normal or low weight	340 (62.85%)	88 (17.53%)	
Overweight	119 (22.00%)	118 (23.51%)	
Obesity	82 (15.16%)	296 (58.96%)	
**Smoking status**			0.852
Every day	67 (12.38%)	54 (10.76%)	
Some days	18 (3.33%)	16 (3.19%)	
Not at all	51 (9.43%)	51 (10.16%)	
Missing data	405 (74.86%)	381 (75.90%)	
**Drinking status**			0.821
None or light drinker	113 (20.89%)	112 (22.31%)	
Moderate drinker	271 (50.09%)	246 (49.00%)	
Heavy drinker	7 (1.29%)	4 (0.80%)	
Missing data	150 (27.73%)	140 (27.89%)	
**Total cholesterol (mg/dL)**	171.60 ± 34.88	175.34 ± 34.98	0.061
**HDL (mg/dL)**	62.46 ± 14.74	51.03 ± 12.63	< 0.001
**LDL (mg/dL)**	95.28 ± 29.43	104.06 ± 29.35	< 0.001
**Triglyceride (mg/dL)**	69.39 ± 42.58	101.07 ± 66.25	< 0.001
**Fasting blood glucose (mg/dL)**	90.93 ± 7.44	101.78 ± 24.35	< 0.001
**Insulin (uU/mL)**	6.05 ± 2.10	20.30 ± 19.18	< 0.001
**TyG index**	7.91 ± 0.51	8.36 ± 0.62	< 0.001
**TyG-BMI index**	194.68 ± 48.12	277.75 ± 80.04	< 0.001
**Hypertension**			0.002
Yes	39 (7.21%)	65 (12.95%)	
No	502 (92.79%)	437 (87.05%)	
**Diabetes**			< 0.001
Yes	2 (0.37%)	22 (4.38%)	
No	537 (99.26%)	472 (94.02%)	
Borderline	2 (0.37%)	8 (1.59%)	
**Infertility**			< 0.001
No	513 (94.82%)	435 (86.65%)	
Yes	28 (5.18%)	67 (13.35%)	

Values are presented as mean ± standard deviation or n (%)

Abbreviations: RIP, ratio of family income to poverty; BMI, body mass index; HDL, high-density lipoprotein; LDL, low-density lipoprotein; HOMA-IR, homeostasis model assessment of insulin resistance; TyG, triglyceride glucose index; TyG-BMI, triglyceride glucose-body mass

### The association between different IR surrogates and female infertility

**Fig. 2:**
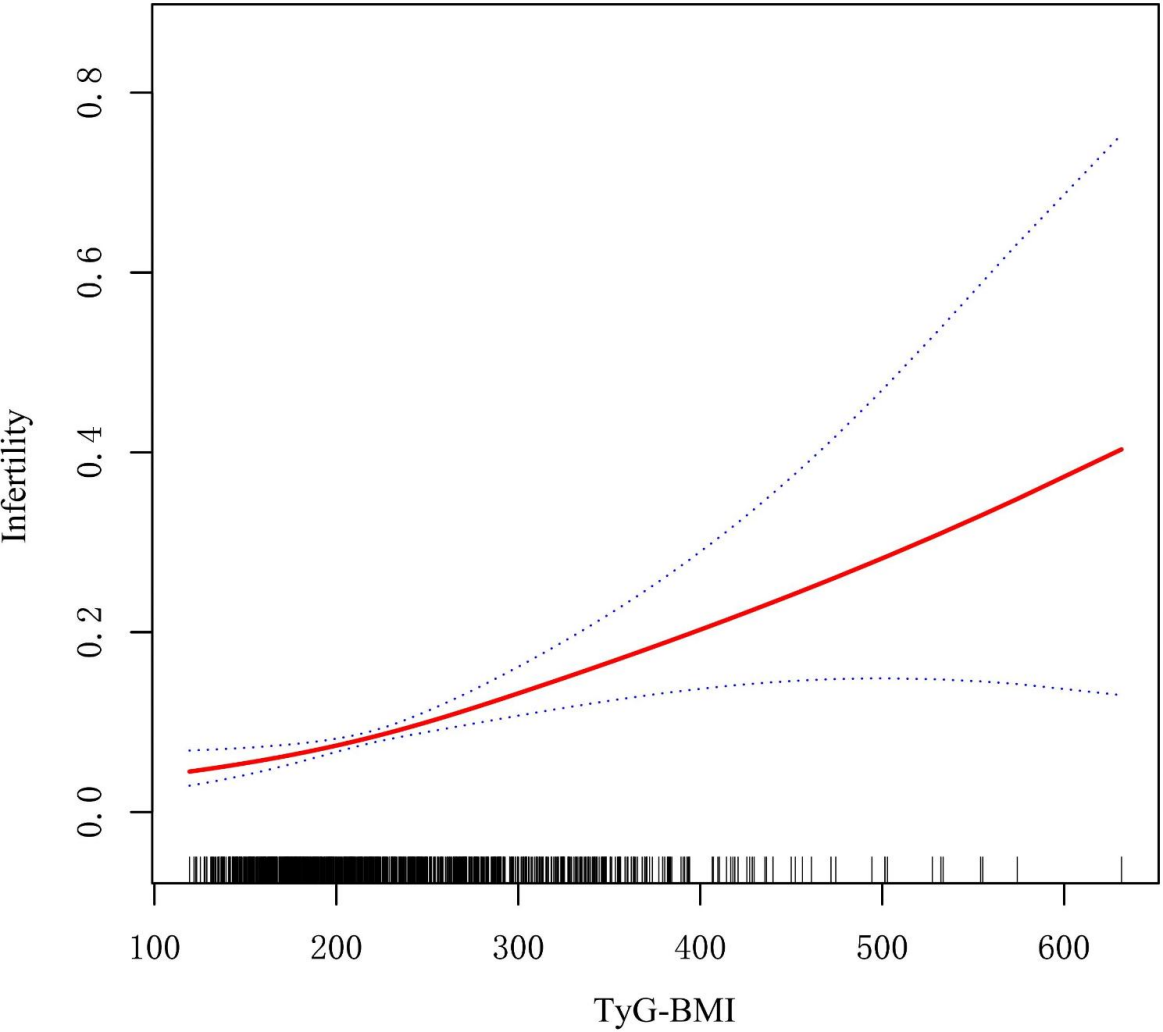
Restricted cubic spline fitting for the association between TyG-BMI with female infertility

Table 2 shows the ORs and 95% CIs of the association between different IR surrogates and female infertility in the three regression models. In both Model I and Model II, HOMA-IR index, TyG index and TyG-BMI index showed positive correlations with female infertility (all P < 0.05). However, in the fully adjusted Model III we found that HOMA-IR index and TyG index did not correlate with female infertility (P > 0.05), while TyG-BMI index still showed a robust positive correlation with female infertility (OR = 1.01, 95% CI: 1.00, 1.01; P < 0.0001). Based on these results, we further examined the differences in the risk of infertility between the different TyG-BMI tertile groups. It was found that in all three models, the ORs of the TyG-BMI group T3 (≥ 255.55) was significantly different compared to the group T1 (< 185.31) (Model I: OR = 4.15, 95% CI: 2.33, 7.38; Model II: OR = 3.56, 95% CI: 1.98, 6.39; Model III: OR = 3.02, 95% CI: 1.62, 5.60), suggesting the risk of female infertility is significantly increased with higher TyG-BMI index. Additionally, the Fig. 2 also suggests a stable positive correlation between TyG-BMI and female infertility.

**Table 2:** Multivariate logistic regression analysis of different insulin resistance surrogates with infertility

Exposure	Model I OR(95%CI) P-value	Model II OR(95%CI) P-value	Model III OR(95%CI) P-value
HOMA-IR index	1.03 (1.00, 1.07) 0.0256	1.04 (1.01, 1.07) 0.0101	1.02 (0.99, 1.06) 0.2440
TyG index	2.00 (1.43, 2.78) < 0.0001	1.80 (1.29, 2.52) 0.0006	1.37 (0.93, 2.01) 0.1141
TyG-BMI index	1.01 (1.00, 1.01) < 0.0001	1.01 (1.00, 1.01) < 0.0001	1.01 (1.00, 1.01) < 0.0001
TyG-BMI index tertile			
T1 (< 185.31)	1.0	1.0	1.0
T2 (≥ 185.31, < 255.55)	1.34 (0.69, 2.61) 0.3946	1.16 (0.59, 2.28) 0.6641	1.10 (0.56, 2.19) 0.7797
T3 (≥ 255.55)	4.15 (2.33, 7.38) < 0.0001	3.56 (1.98, 6.39) < 0.0001	3.02 (1.62, 5.60) 0.0005
P-value for trend	< 0.0001		

Model I adjust for: None

Model II adjust for: Age

Model III adjust for: Age, Marital status, Education level, LDL, Hypertension, Diabetes

**Fig. 3:**
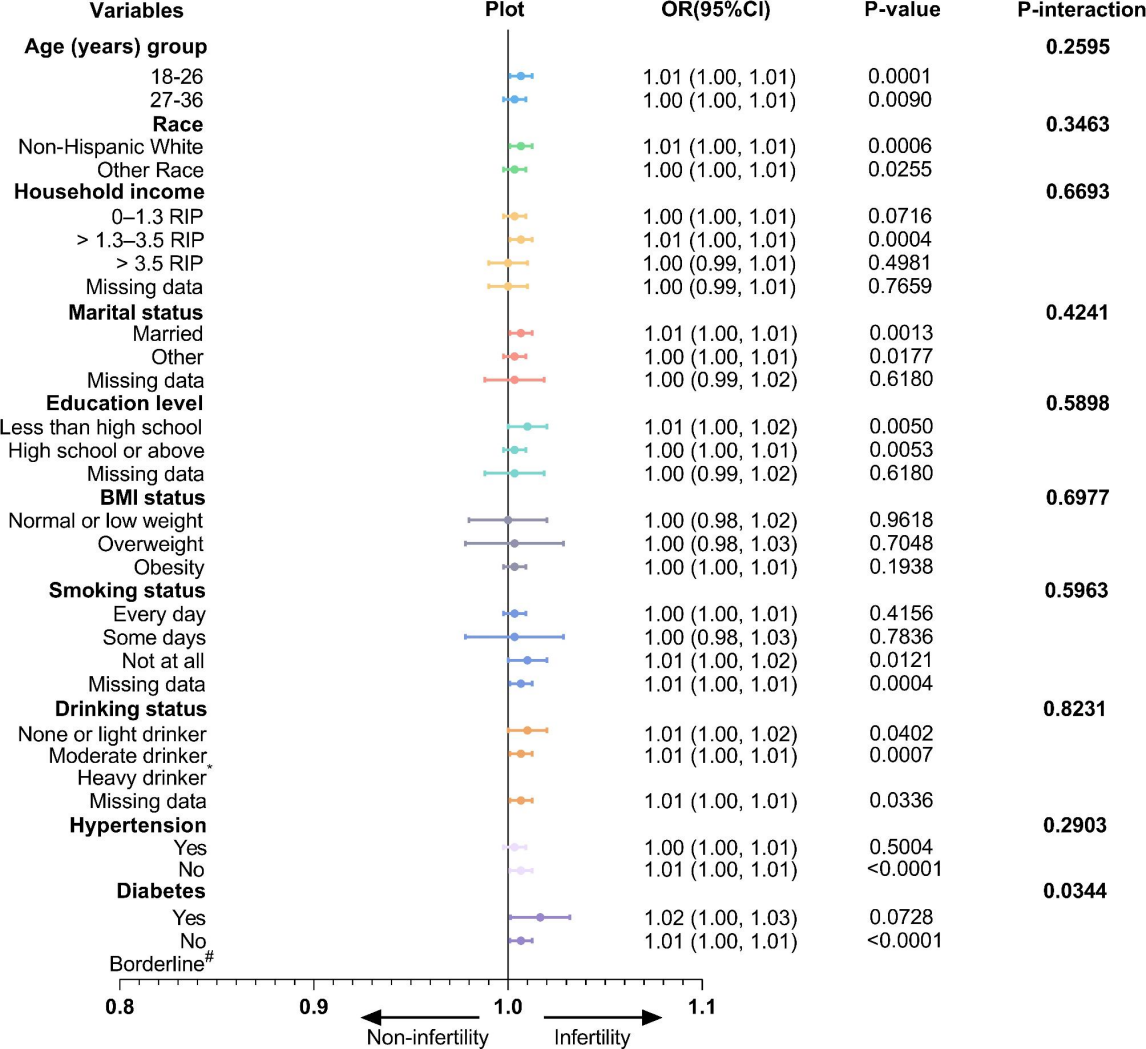
Stratified associations between TyG-BMI index and female infertility according to baseline characteristics

Then, we performed stratified analysis to assess the effect of TyG-BMI on infertility. As shown in Fig. 3, the association between TyG-BMI and infertility was similar in most of the stratified population (P-interaction > 0.05). In addition, the results of the univariate logistic regression analysis of infertility were shown in Table S1.

### Comparison of different IR surrogates in predicting insulin resistance and infertility

**Fig. 4:**
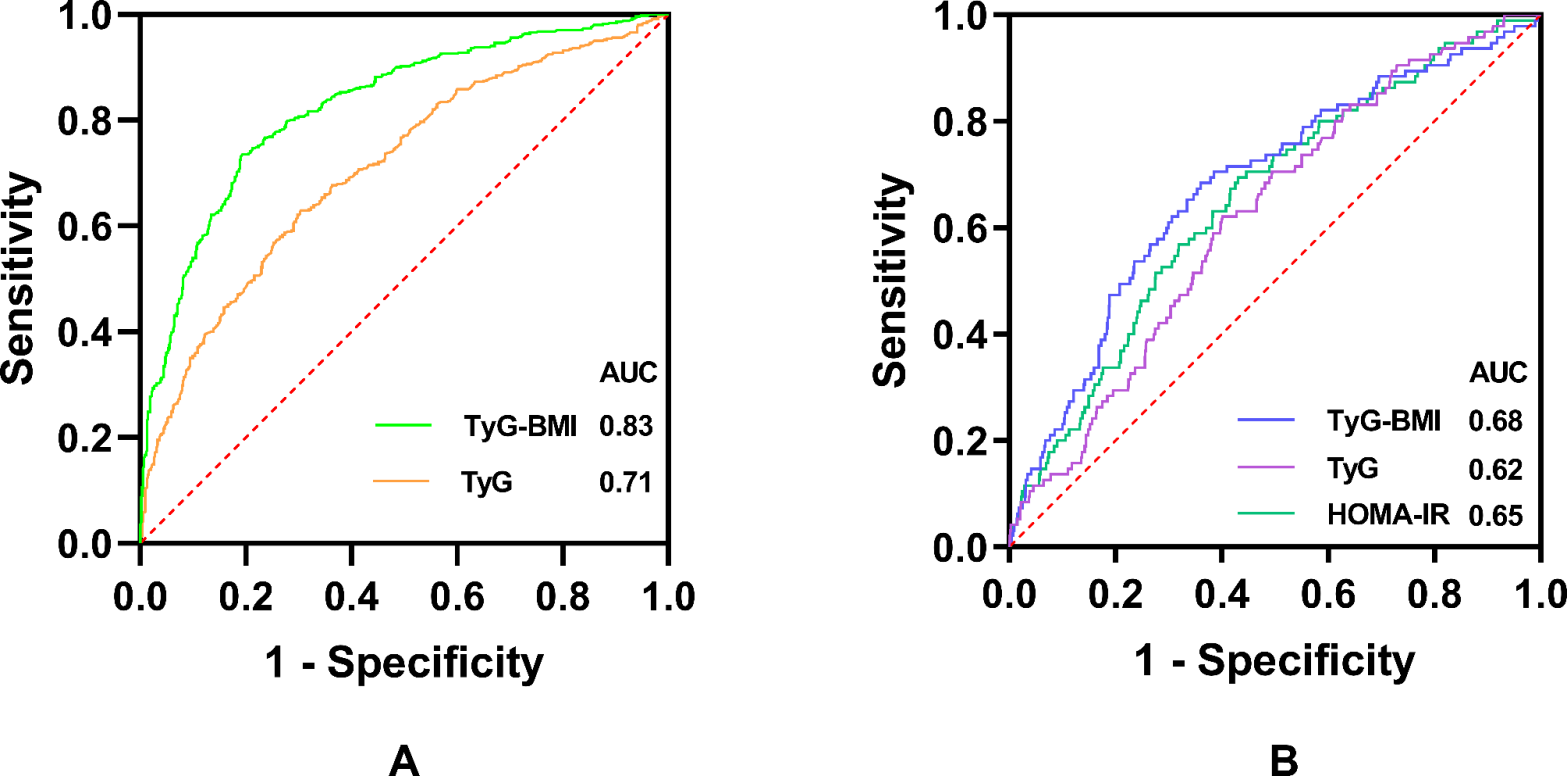
(**A**) ROC curves for different surrogates to predict insulin resistance. (**B**) ROC curves for different surrogates to predict female infertility

The results of the ROC curves were shown in Table 3; Fig. 4. The AUCs of TyG-BMI index and TyG index in predicting insulin resistance (defined as HOMA-IR ≥ 2.2) were 0.83 (95% CI: 0.80, 0.85) and 0.71 (95% CI: 0.68, 0.74), respectively, suggesting that TyG-BMI index was significantly better than TyG index in predicting insulin resistance (P < 0.0001). As for the infertility, the AUCs of TyG-BMI index, TyG index and HOMA-IR index were 0.68 (95% CI: 0.62, 0.74) vs. 0.62 (95% CI: 0.57, 0.68) vs. 0.65 (95% CI: 0.60, 0.71). Although the AUC of TyG-BMI was the largest, there was no statistical difference between the AUCs of the different surrogates (P > 0.05).

**Table 3:** Comparison of ROC curves for different surrogates to predict insulin resistance and infertility

Objects/Surrogates	Cutoff (Sensitivity, Specificity)	AUC (95% CI)	P-value
**Insulin resistance**			
TyG index	8.12 (0.63, 0.70)	0.71 (0.68, 0.74)	< 0.0001
TyG-BMI index	226.48 (0.74, 0.81)	0.83 (0.80, 0.85)	
**Infertility**			
HOMA-IR index	2.34 (0.69, 0.57)	0.65 (0.60, 0.71)	> 0.05
TyG index	8.19 (0.62, 0.60)	0.62 (0.57, 0.68)	
TyG-BMI index	242.18 (0.68, 0.64)	0.68 (0.62, 0.74)	

AUC, area under the curve; CI, confidence interval

Z-test was used to compare tatistically significant differences between AUCs

## Discussion

In this cross-sectional study, we explored the relationship between different IR surrogates and female infertility in the general population. Our study revealed that high levels of TyG-BMI were positively associated with female infertility in US reproductive-aged females. The association was also similar in most of the subgroup participants. However, different IR surrogates did not show variability in predicting infertility. To our knowledge, this was the first study to explore the relationship between different IR surrogates and female infertility in the general population.

Obesity is often strongly associated with glucose intolerance and insulin resistance, these risk factors often collectively referred to as the metabolic syndrome, which has been inferred to have negative impact on fertility and pregnancy [21]. A large 15-year population-based cohort study from Australia suggested that higher BMI is positively associated with infertility problems [22]. Similarly, Zhu et al. conducted a cross-sectional study that included 3624 participants and reveals that BMI at extreme levels tended to be infertility [10]. In addition, PCOS is one of the major causes of female infertility, while many abnormalities of the metabolic syndrome overlap with those of PCOS, and current studies suggest that insulin resistance and glucose intolerance are the same pathogenesis for both [23]. In this context, it is widely believed that IR is also the main pathophysiological mechanism and central aspect that leads to infertility in PCOS [24–26]. Furthermore, IR also has a negative impact on assisted reproductive technology (ART) outcomes. A secondary analysis of an ART multicenter randomized trial conducted by He et al. [27] found that patients with metabolic syndrome have longer duration of infertility compared to those without metabolic syndrome, and that metabolic syndrome is negatively associated with cumulative live birth rate. Another prospective cohort study from China identified that IR is associated with decreased percentage of mature oocytes and poor embryo quality in lean and infertile women without PCOS [28]. Similarly, Song et al. [29] retrospectively analyzed data from 329 women receiving IVF and found that higher HOMA-IR and BMI result in a significant decrease of clinical pregnancy rate, regardless of whether PCOS is combined.

Different mechanisms are thought to contribute to the negative effects of IR on female reproductive function. Firstly, IR may affect oocyte quality by reducing mitochondrial function, which is the main source of energy production and the major generator of reactive oxygen species (ROS) in the oocyte cytoplasm, and is closely related to oocyte quality. A study from OU et al. [30] using the mouse model found that maternal IR enhanced oxidative stress in follicles after controlled ovarian hyperstimulation and disrupted mitochondrial function in mouse oocytes. Besides, mitochondrial damage produces a large amount of ROS, which induces the release of inflammatory factors [such as TNF-α, interleukin 1β (IL-1β) and IL-6] and disrupts pancreatic β-cell function, further aggravating insulin resistance. Eventually, a vicious cycle is formed between IR, mitochondrial damage and inflammation [31]. Secondly, IR affects the energy metabolism of oocytes. Glucose transporter (GLUT4) is responsible for cellular energy supply, several studies have suggested that decreased GLUT4 expression in PCOS patients with IR reduces glucose uptake and utilization by ovarian granulosa cells and finally negatively affects oocyte quality [32, 33]. In addition, hyperandrogenemia is thought to play an important role in PCOS leading to infertility, and it has been demonstrated that hyperinsulinemia acts synergistically with LH on ovarian follicular membrane cells to increase cytochrome P450c17 activity, resulting in increased androgen production [34]. Wu et al. [35] revealed that specific knockdown of insulin receptor (INSR) on ovarian membrane cells results in decreased androgen levels and increased fertility in mice. At last, besides affecting oocyte quality, IR affects endometrial tolerance through various pathways, such as energy metabolism, AMP-activated protein kinase (AMPK), insulin receptor substrate (IRS)/PI3K/Akt pathway, and chronic inflammation, which in turn affects female reproductive function [36–38].

The gold standard for assessing metabolic insulin resistance in vivo is the hyperinsulinemic-euglycemic clamp (HIEC) [39, 40]. This technique quantitatively assesses the effect of insulin on systemic glucose uptake by infusing the required dose of insulin and maintaining normoglycemia using variable glucose infusion, in which the infusion rate is adjusted according to frequent arterialized glucose measurements and the negative feedback [39, 40]. Due to the complexity and cost of HIEC, there is a desire to use clinically accessible fasting parameters of glucose homeostasis as an alternative means to confirm the diagnosis of IR, and these measures include homeostatic model assessment [19], TyG index [16], and TyG-BMI index [17]. In our study, the association of TyG-BMI with female infertility was found to be more superior than the other two surrogates, and this superiority was similar in other IR-related diseases. In a prospective cohort study comparing the association between different IR surrogates and diabetes, TyG-BMI was found to have the strongest association with diabetes in patients with impaired fasting glucose and the best predictive efficacy [17]. A cross-sectional study from Korea that included 11,149 participants also found that the TyG-BMI index is higher than other parameters in predicting IR [41]. Similarly, WANG et al. revealed that the association between TyG-BMI index and hyperuricemia in non-diabetic patients is similarly superior compared to other IR surrogates by analyzing data from NHANES [42]. The mechanism for the better predictive ability of TyG-BMI index is not yet clear, probably because compared with HOMA-IR index or TyG index, TyG-BMI index contains not only abnormal glucose metabolism and defective fatty acid metabolism, but it also includes BMI, one of the obesity indices, to improve its diagnostic ability.

However, this study also has some limitations. First, due to limitations of the NHANES database, the definition of the outcome variable female infertility comes from self-reporting. Although self-reported infertility is a useful measurement method, it may not be very accurate in some cases. For example, women who are planning to conceive for less than a year but have already sought medical help may be included. And the various definitions of infertility (i.e., medical records or calendar-derived time taken trying to conceive) may affect the prevalence of measured infertility [43, 44]. Further research needs to consider the impact of different definitions. Secondly, this was a cross-sectional study and was not compared with a cohort of ethnically and age-matched fertile female, so we could not obtain a causal relationship. Finally, only female participants aged between 18 and 36 years were included in this study, and the sample size is not large, which may serve as a potential source of bias, so explorations of wider population should be further developed.

## Conclusions

In a nationally representative sample of US adult females, the HOMA-IR index and TyG index not show an association with female infertility, while the TyG-BMI index is found to have a stable and strong positive association with female infertility, which provides new insights into the prevention and management of female infertility. However, different IR surrogates not show variability in their ability to predict infertility. Future cohort studies with a wider population are needed to validate this relationship.

### Electronic supplementary material

Below is the link to the electronic supplementary material.

Supplementary Material 1: Table S1 Univariate logistic regression analysis of different insulin resistance surrogates with infertility

## Data Availability

Data set used in this study will be available from corresponding author on reasonable request.
