# Supplementary material for: Association between different insulin resistance surrogates and infertility in reproductive-aged females
Source: BMC Public Health. 2023 Oct 12;23:1985. doi: 10.1186/s12889-023-16813-2 (PMC10568938; doi:10.1186/s12889-023-16813-2)
Supplement: Supplementary file 1 — Supplementary Material 1: Table S1 Univariate logistic regression analysis of different insulin resistance surrogates with infertility [file 12889_2023_16813_MOESM1_ESM.docx]

**Table S1** Univariate logistic regression analysis of different insulin resistance surrogates with infertility

| Variables | OR(95%CI) | P-value |
| --- | --- | --- |
| **Age (years)** | 1.08 (1.04, 1.12) | 0.0001 |
| **Race** |  |  |
| Non-Hispanic White | 1.0 |  |
| Other Race | 0.78 (0.51, 1.21) | 0.2712 |
| **Marital status** |  |  |
| Married | 1.0 |  |
| Other | 0.51 (0.33, 0.78) | 0.0022 |
| Missing data | 0.12 (0.04, 0.38) | 0.0004 |
| **Education level** |  |  |
| Less than high school | 1.0 |  |
| High school or above | 0.70 (0.40, 1.26) | 0.2354 |
| Missing data | 0.13 (0.04, 0.45) | 0.0014 |
| **Household income** |  |  |
| 0–1.3RIP | 1.0 |  |
| > 1.3–3.5 RIP | 1.04 (0.65, 1.66) | 0.8752 |
| > 3.5 RIP | 0.54 (0.27, 1.05) | 0.0710 |
| Missing data | 0.77 (0.31, 1.89) | 0.5670 |
| **BMI status** |  |  |
| Normal or low weight | 1.0 |  |
| Overweight | 1.56 (0.79, 3.09) | 0.2041 |
| Obesity | 4.06 (2.38, 6.94) | <0.0001 |
| **Smoking status** |  |  |
| Every day | 1.0 |  |
| Some days | 0.55 (0.15, 2.00) | 0.3679 |
| Not at all | 0.91 (0.43, 1.94) | 0.8072 |
| Missing data | 0.47 (0.27, 0.83) | 0.0095 |
| **Drinking status** |  |  |
| None or light drinker | 1.0 |  |
| Moderate drinker | 1.37 (0.78, 2.39) | 0.2689 |
| Heavy drinker | 6.57 (1.76, 24.59) | 0.0052 |
| Missing data | 0.76 (0.39, 1.50) | 0.4298 |
| **Total cholesterol (mg/dL)** | 1.00 (0.99, 1.00) | 0.6073 |
| **HDL (mg/dL)** | 0.95 (0.94, 0.97) | <0.0001 |
| **LDL (mg/dL)** | 1.00 (1.00, 1.01) | 0.4611 |
| **Triglyceride (mg/dL)** | 1.00 (1.00, 1.01) | 0.0065 |
| **Fasting blood glucose (mg/dL)** | 1.02 (1.01, 1.03) | 0.0002 |
| **Insulin (uU/mL)** | 1.01 (1.00, 1.02) | 0.0170 |
| **HOMA-IR index** | 1.03 (1.00, 1.07) | 0.0256 |
| **TyG index** | 2.00 (1.43, 2.78) | <0.0001 |
| **TyG-BMI index** | 1.01 (1.00, 1.01) | <0.0001 |
| **Hypertension** |  |  |
| Yes | 1.0 |  |
| No | 0.46 (0.26, 0.82) | 0.0081 |
| **Diabetes** |  |  |
| Yes | 1.0 |  |
| No | 0.10 (0.05, 0.24) | <0.0001 |
| Borderline | 0.30 (0.05, 1.69) | 0.1709 |

Abbreviations: RIP, ratio of family income to poverty; BMI, body mass index; HDL, high-density lipoprotein; LDL, low-density lipoprotein; HOMA-IR, homeostasis model assessment of insulin resistance; TyG, triglyceride glucose index; TyG-BMI, triglyceride glucose-body mass
